# Effects of Spraying Mn_3_O_4_ Nanozymes at Different Concentrations on the Physiological Characteristics and Yield of Wheat Flag Leaves Under High-Temperature Stress During the Filling Stage

**DOI:** 10.3390/plants15203096

**Published:** 2026-10-10

**Authors:** Qian Yang, Chuanxin Chen, Junjie Lei, Shihui Nie, Juan Li, Yuting Hou, Wenxiu Xu, Yongqiang Zhang

**Affiliations:** 1 College of Agriculture, Xinjiang Agricultural University, Urumqi 830052, China; 17361238862@163.com; 2 Institute of Crop Research, Xinjiang Academy of Agricultural Sciences, Key Laboratory of Desert–Oasis Crop Physiology, Ecology and Cultivation, Urumqi 830091, China; lixp618@163.com (C.C.); leijunjie@sohu.com (J.L.); nieshihui@hotmail.com (S.N.); l_juan@yeah.net (J.L.); yuthou@163.com (Y.H.)

**Keywords:** wheat, high-temperature stress during grain filling, Mn_3_O_4_ nanozymes, physiological characteristics, yield

## Abstract

Climate change-driven high-temperature stress has become a major threat to global wheat production, and developing effective strategies to ensure heat-resilient, stable-yield cultivation is an urgent scientific priority. Mn_3_O_4_ nanozymes, a newly developed type of nanozyme, have not yet been tested for improving wheat heat tolerance. This study investigated the physiological mechanisms by which Mn_3_O_4_ nanozymes alleviate high-temperature stress during wheat grain filling. A polyethylene film greenhouse simulated a high-temperature environment, increasing the average canopy temperature of wheat by 2–3 °C. Under stress, plants were sprayed with distilled water (M0) or with Mn_3_O_4_ nanozymes at 100 (M1), 150 (M2), or 200 mg·L^−1^ (M3), while an additional control (CK) was sprayed with distilled water under natural conditions. Compared with the CK, high-temperature stress significantly disrupted chloroplast structure, altered stomatal morphology, and reduced SPAD values, flag leaf area, and photosynthetic parameters in flag leaves, ultimately leading to a decrease in thousand-kernel weight (down 4.86%) and wheat yield (down 21.24%); in contrast, under high-temperature stress, Mn_3_O_4_ nanozyme treatment resulted in milder damage to chloroplast membrane structure; the thylakoid membrane system exhibited a slightly relaxed state overall, and stomatal aperture remained favorable. This treatment slowed the decline in photosynthetic capacity and significantly enhanced antioxidant enzyme activity, thereby mitigating the impact on yield. Among these, the M1 treatment yielded the best results, with thousand-kernel weight and yield increasing by an average of 2.62% and 18.26%, respectively, compared to M0; however, this mitigating effect diminished as the concentration rose. Compared with the M0 treatment, M2 and M3 treatments produced average yield increases of 9.04% and 5.34%, respectively. Therefore, Mn_3_O_4_ nanozymes at an appropriate concentration can mitigate the adverse effects of high-temperature stress on wheat at multiple levels, thereby effectively alleviating yield losses during the grain-filling stage.

## 1. Introduction

Wheat (*Triticum aestivum* L.) is a vital food crop in China, providing a crucial source of nutrients for the population; stabilizing and expanding production is essential to ensure food security [1,2]. However, as industrialization progresses and human activities intensify, global warming is becoming increasingly severe, leading to more frequent extreme heat events and a significantly increased risk of high-temperature stress in wheat. Studies have found that for every 1 °C increase in global average temperature, wheat yields will decline by approximately 4.1% to 6.4% [3], posing a serious threat to food security. Xinjiang is an arid and semi-arid region. As the global greenhouse effect intensifies, the “warming and moistening” climate change in Xinjiang has become increasingly pronounced, causing wheat in the grain-filling stage to experience premature senescence and abnormal maturation due to rising temperatures, which affects grain filling and quality formation and seriously threatens the safe production of wheat [4,5]. Therefore, actively exploring effective measures to mitigate high-temperature stress and establishing a stress-tolerant, stable-yield cultivation technology system are scientific issues that urgently require attention.

Crops respond to high-temperature stress in complex and multidimensional ways. High temperatures can disrupt the ultrastructure of organelles such as chloroplasts, leading to disorganized thylakoid membrane arrangements and loosened stroma layers; they also affect stomatal morphology and opening and closing, directly impacting light absorption and conversion [6,7,8]. Reactive oxygen species (ROS) are an important class of signaling molecules within cells. Under normal physiological conditions, ROS production and clearance within cells maintain a dynamic equilibrium [9,10]. Under high-temperature stress, plants undergo oxidative damage that leads to electrolyte leakage, triggering metabolic disorders and excessive ROS accumulation, which in turn causes membrane lipid peroxidation and damages the photosynthetic membrane system [11,12]. As a result, photosynthesis is inhibited, photosynthetic pigment content decreases, photosynthetic efficiency declines, and the synthesis of photosynthetic assimilates is reduced [13,14]. High temperatures can also disrupt the physiological balance in wheat plants, accelerate aging, shorten the grain-filling period, and reduce grain filling, ultimately affecting both yield and quality [15,16,17]. Therefore, exploring effective mitigation strategies to preserve leaf structural integrity and photosynthetic capacity during the filling stage is a critical approach to minimizing losses under high temperatures.

Using exogenous substances to regulate crop growth is a key measure for improving crop tolerance to high temperatures. In modern agriculture, nanomaterials offer broad application prospects; they can promote sustainable agricultural development and enhance crop yield and quality while significantly reducing adverse impacts on the ecological environment [18,19]. Nanomaterials are materials in which at least one dimension in three-dimensional space ranges from 1 to 100 nm [20]. Nanozymes are a class of nanomaterials that possess the natural catalytic activity of enzymes. Compared with natural enzymes, nanozymes can often maintain catalytic activity under high-temperature, acidic, or alkaline conditions and offer advantages such as low cost and ease of storage and transport [21]. Mn_3_O_4_ nanozymes are a type of nanozyme that has been newly developed in recent years and, thanks to their unique properties, have been widely applied in various fields. Studies have demonstrated that Mn_3_O_4_ nanozymes exhibit catalytic activities analogous to superoxide dismutase (SOD) and catalase (CAT). Relevant studies have shown that Mn_3_O_4_ nanozymes can enhance the tolerance of crops such as soybean, rapeseed, and cotton to salt–alkali stress and drought stress; their mechanisms primarily involve maintaining ROS homeostasis and regulating K^+^/Na^+^ balance [22,23,24,25]. Furthermore, existing research on Mn_3_O_4_ nanozymes has primarily focused on application routes such as seed soaking; to date, no publicly available studies have been reported on the application of Mn_3_O_4_ nanozymes via foliar spraying under high-temperature stress conditions in wheat, and there is a particular lack of research on the use of this nanozyme to alleviate high-temperature stress during the grain-filling stage of wheat. Consequently, it remains unclear whether foliar application of Mn_3_O_4_ nanozymes can alleviate high-temperature stress in wheat.

Therefore, this study used field greenhouses to simulate high-temperature stress. Spraying treatments with different concentrations of Mn_3_O_4_ nanozymes were applied to observe their effects on the following aspects: (1) the ultrastructure of chloroplasts and stomatal morphology in flag leaves; (2) flag leaf senescence; and (3) yield. This study aims to elucidate the physiological mechanisms by which Mn_3_O_4_ nanozymes alleviate high-temperature stress during the grain-filling stage of wheat, and to screen for optimal spray application concentrations, thereby providing a theoretical foundation and technical support for advancing nano-agriculture and enhancing wheat yield.

## 2. Results

### 2.1. Effects of Mn_3_O_4_ Nanozymes on SPAD Value and Flag Leaf Area Under High-Temperature Stress During the Filling Stage

**Figure 1:**
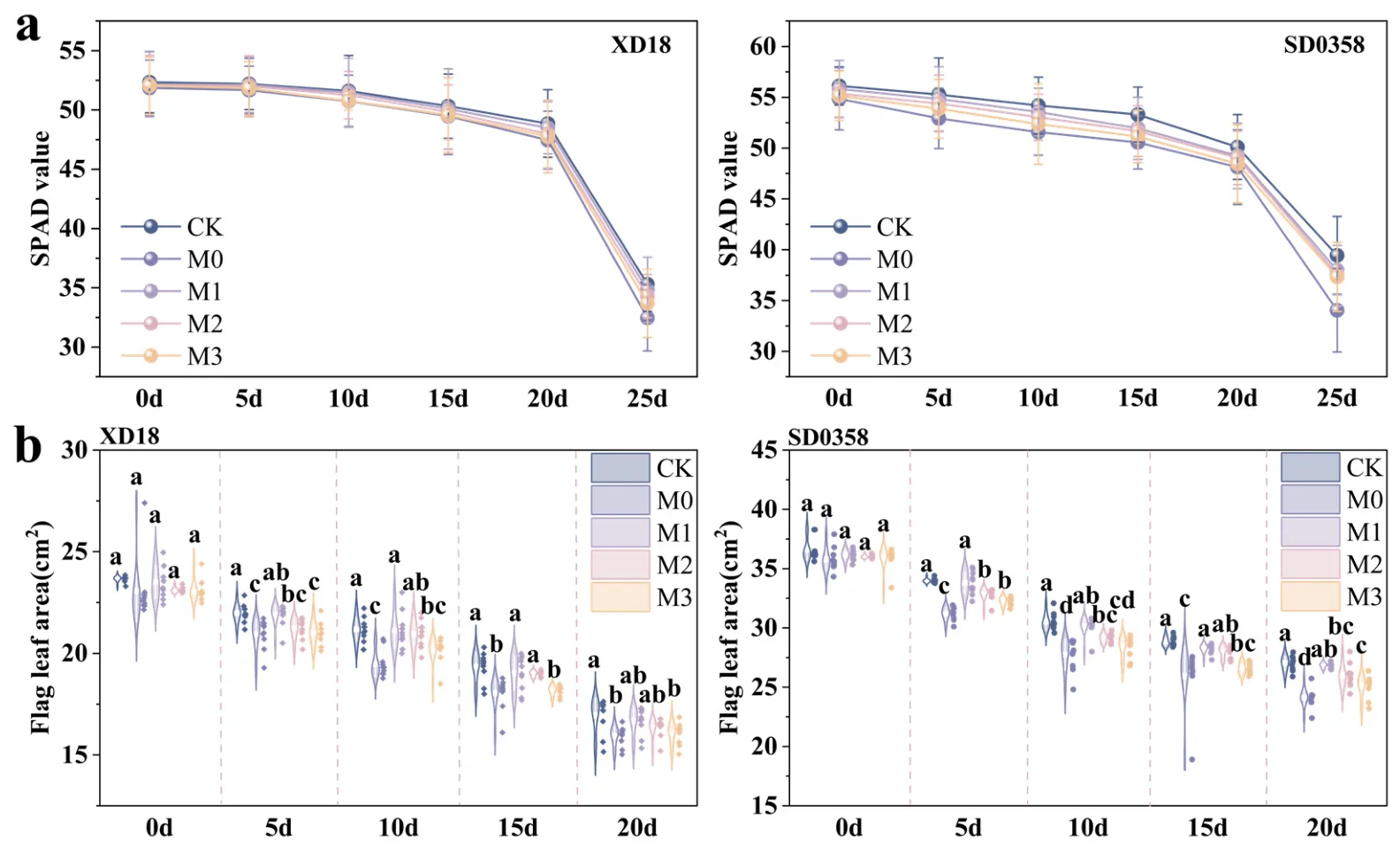
The effect of Mn_3_O_4_ nanozymes on SPAD value (**a**) and flag leaf area (**b**) under high-temperature stress during the filling stage. CK, distilled water under natural temperature conditions; M0, distilled water under high-temperature conditions; M1, 100 mg·L^−1^ nanomimetic enzymes under high-temperature conditions; M2, 150 mg·L^−1^ nanomimetic enzymes under high-temperature conditions; M3, 200 mg·L^−1^ nanomimetic enzymes under high-temperature conditions; d, days after treatment. The error bars represent the standard deviation, and different lowercase letters denote significant differences.

Both SPAD value and flag leaf area declined as treatment duration increased (Figure 1a). From the fifth day of treatment, differences emerged among the treatment groups, and flag leaf area also varied significantly (Figure 1b). Further analysis showed that, as treatment duration increased under high-temperature stress, the decline in SPAD value and flag leaf area under the M0 treatment was particularly pronounced, with a decline rate markedly higher than that of the other treatments.

### 2.2. Effects of Mn_3_O_4_ Nanozymes on Photosynthetic Parameters of the Flag Leaf Under High-Temperature Stress During the Filling Stage

**Figure 2:**
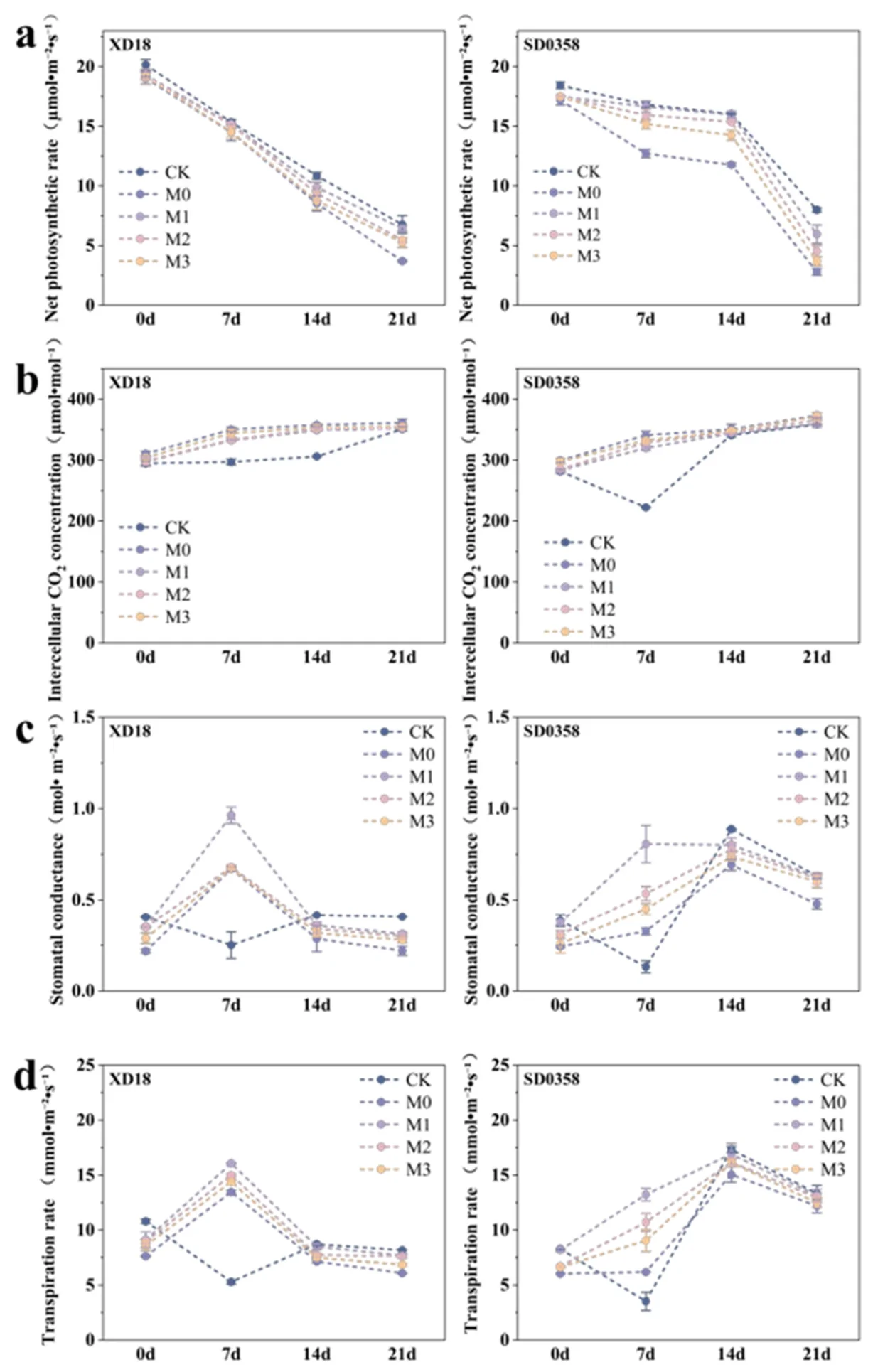
Effects of spraying Mn_3_O_4_ nanozymes on photosynthetic parameters of the flag leaf under high-temperature stress during the filling stage. (**a**) Net photosynthetic rate (Pn); (**b**) Intercellular CO_2_ concentration (Ci); (**c**) Stomatal conductance (Gs); (**d**) Transpiration rate (Tr). The error bars represent the standard deviation.

As shown in Figure 2, the Pn values for all treatments of both varieties generally decreased as the growth process progressed. Under high-temperature stress, Pn values for all treatments of both varieties were lower than in the CK; however, Pn values increased in treatments sprayed with Mn_3_O_4_ nanozymes, with the M1 treatment showing the greatest improvement. Ci, by contrast, showed the opposite pattern: under high-temperature stress, Ci values in all treatments were higher than in the CK, whereas Ci decreased in all treatments sprayed with Mn_3_O_4_ nanozymes, with the most significant reduction in the M1 treatment. For both varieties, Gs and Tr exhibited complex dynamics—initially increasing, then decreasing—as the treatment period progressed. During the early treatment phase (0–7 days), Gs and Tr in all treatment groups under high-temperature stress were significantly higher than those in the CK. By 14 days after treatment, they were significantly lower than in the CK. Therefore, high-temperature stress significantly inhibits photosynthesis, while applying Mn_3_O_4_ nanozymes may alleviate this inhibition.

### 2.3. Effects of Mn_3_O_4_ Nanozymes on Stomatal Activity in the Flag Leaf Under High-Temperature Stress During the Filling Stage

**Figure 3:**
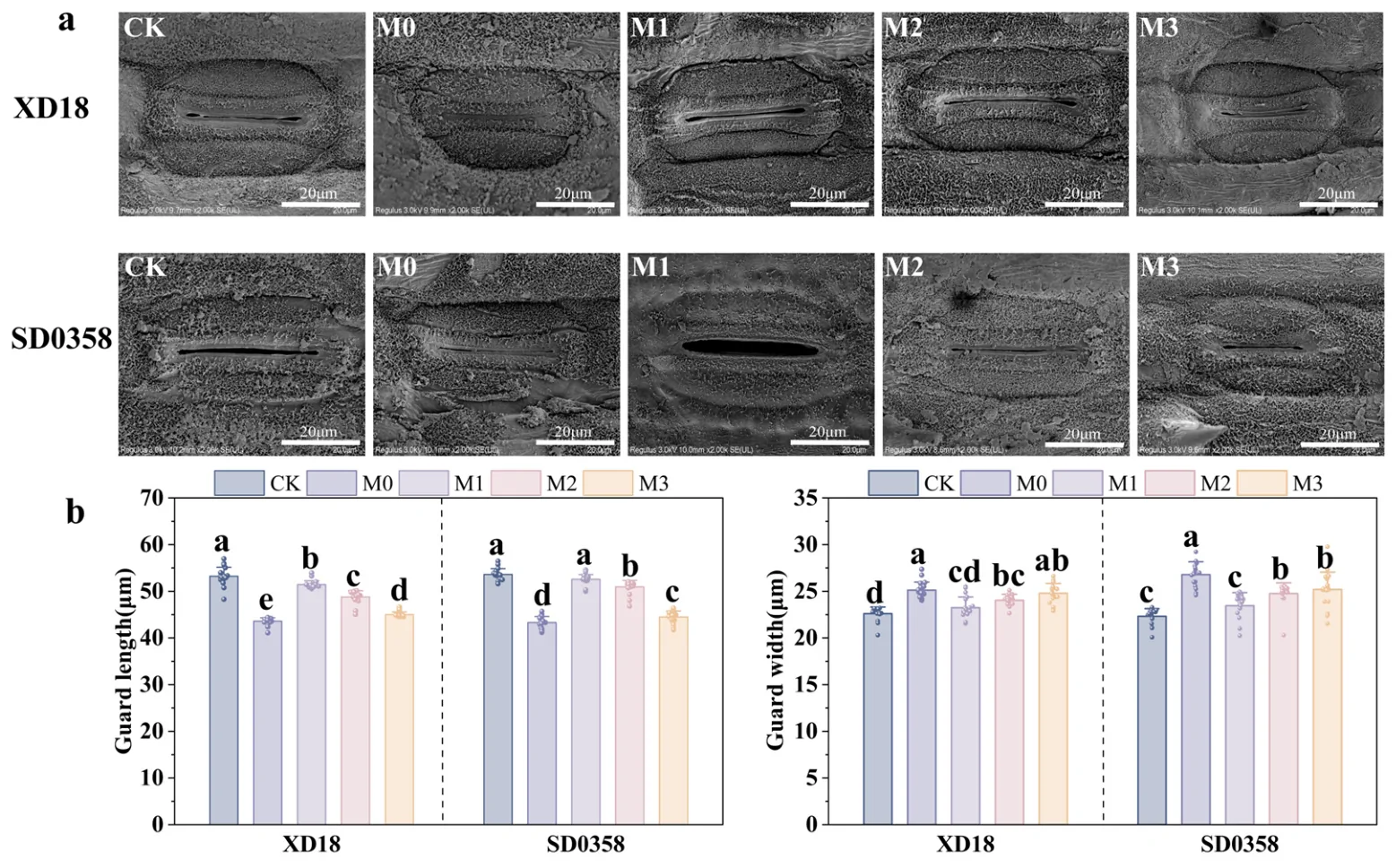
Stomatal morphology of flag leaves under different treatments (scale bar = 20 µm) (**a**) and guard length and guard width (*n* = 19) (**b**). Error bars represent the standard deviation, and different lowercase letters denote significant differences.

Scanning electron microscopy (SEM) images of stomatal morphology in flag leaves from each treatment show that, after high-temperature stress, stomata on flag leaves of both varieties under the M0 treatment were closed compared with CK. In contrast, the stomata in all high-temperature stress treatments treated with Mn_3_O_4_ nanozymes were open (Figure 3a). Among these, M1 showed a larger opening, particularly in SD0358.

Analysis of guard cell length revealed that, under high-temperature stress, the guard cell lengths in all treatments for both varieties were lower than those in the CK under natural conditions; however, the lengths in all treatments sprayed with Mn_3_O_4_ nanozymes were higher than those in the M0 treatment, and guard cell length showed a gradual decrease as the concentration of Mn_3_O_4_ nanozymes increased (Figure 3b). Under high-temperature stress, guard cell width in the M0 treatment for both varieties was wider than in the CK under natural conditions. In all treatments sprayed with Mn_3_O_4_ nanozymes, guard cell width was smaller than in the M0 treatment; however, it increased with increasing Mn_3_O_4_ nanozyme concentration. This fully demonstrates that high-temperature stress significantly reduces guard cell length and increases guard cell width, while spraying Mn_3_O_4_ nanozymes promotes guard cell growth and reduces guard cell width; however, as the concentration of Mn_3_O_4_ nanozymes increases, its effect gradually diminishes.

### 2.4. Effects of Mn_3_O_4_ Nanozymes on the Cellular Structure of Flag Leaves Under High-Temperature Stress During the Filling Stage

**Figure 4:**
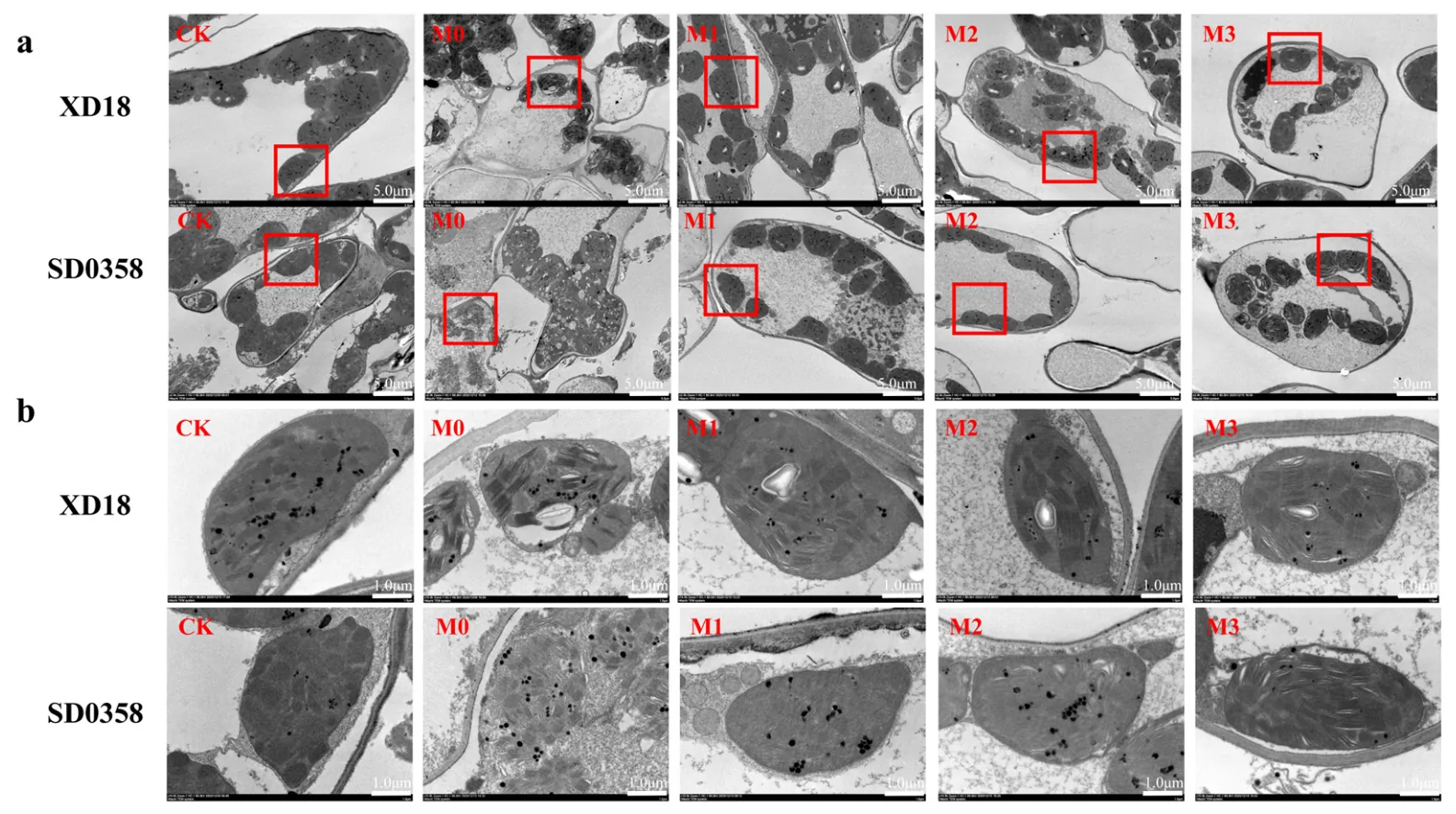
Effects of spraying Mn_3_O_4_ nanozymes on the cellular structure of flag leaves under high-temperature stress during the filling stage. (**a**) Representative chloroplasts (red box). Scale bar = 5.0 µm; (**b**) A high-magnification image of a representative chloroplast. Scale bar = 1.0 µm.

Transmission electron microscopy (TEM) was used to examine the cellular structures of representative flag leaves from each treatment group (Figure 4a,b). To better visualize chloroplast ultrastructure, we selected representative chloroplasts for further observation at higher magnification. Thylakoid membranes in CK-treated samples of both wheat varieties showed a typical stacked structure, with uniform, tightly arranged membranes. The photosynthetic membrane system was intact and undamaged, indicating active function. Following high-temperature stress, chloroplasts in the M0 treatment showed signs of dissolution, with severe structural damage; in contrast, chloroplasts in the M1 treatment exhibited mild damage to the chloroplast membrane structure, and the membrane system as a whole appeared slightly relaxed; under the M2 and M3 treatments, the chloroplast membrane structure was disrupted, with disordered thylakoid membrane stacking, and these abnormalities became more pronounced as the concentration of Mn_3_O_4_ nanozymes increased. This demonstrates that, under high-temperature stress, the chloroplast structure of wheat treated with M1 remained well preserved, indicating good heat resistance.

### 2.5. Effects of Mn_3_O_4_ Nanozymes on the Antioxidant System of the Flag Leaf Under High-Temperature Stress During the Filling Stage

**Figure 5:**
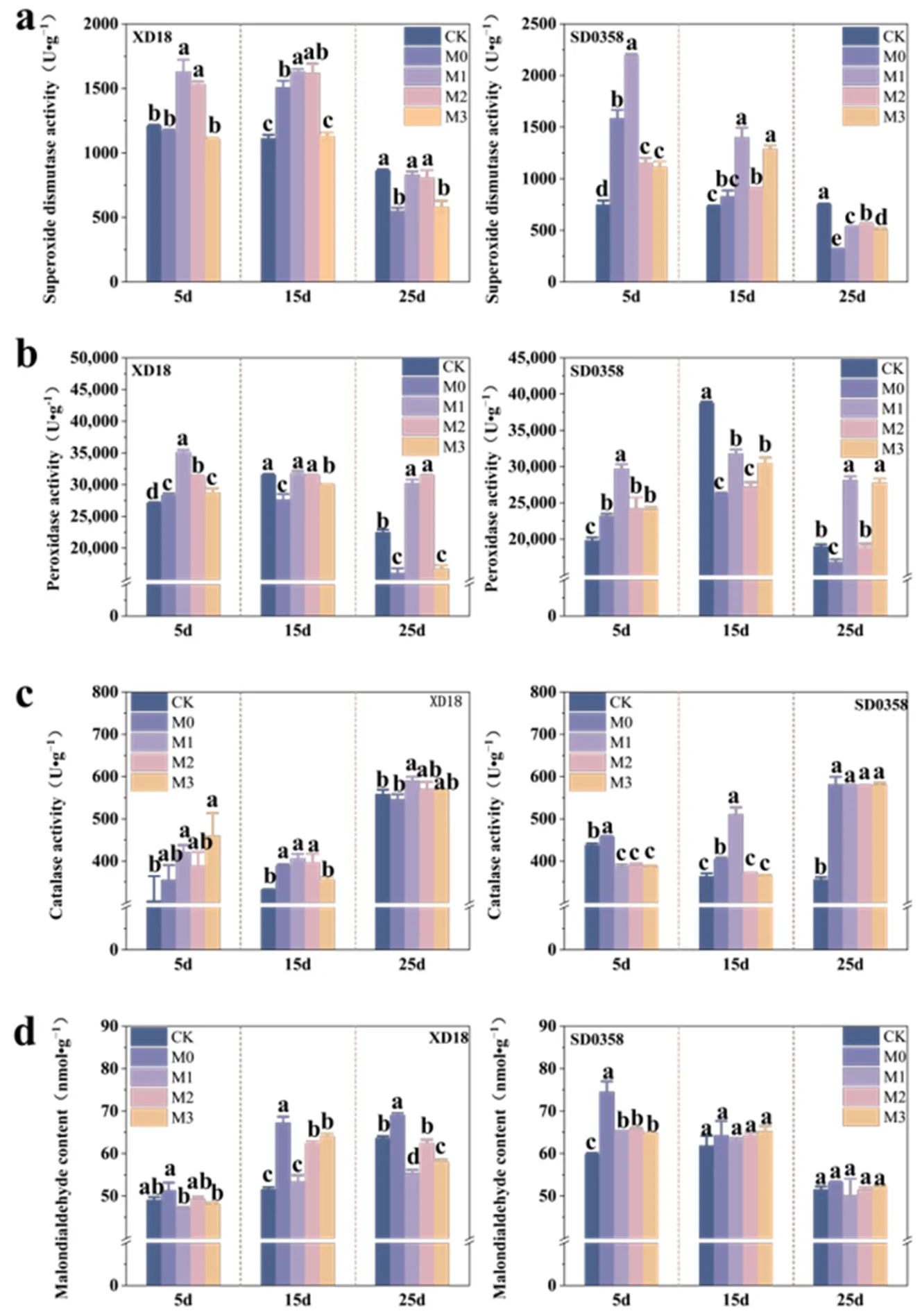
Effects of Mn_3_O_4_ nanozymes on the antioxidant system of flag leaves under high-temperature stress during the filling stage. (**a**) Superoxide dismutase (SOD) activity; (**b**) Peroxidase (POD) activity; (**c**) Catalase (CAT) activity; (**d**) Malondialdehyde (MDA) content. The error bars represent the standard deviation, and different lowercase letters denote significant differences.

On days 5 and 15 of the treatment period, SOD activity in both varieties under high-temperature stress was higher than that in the CK, with the M1 treatment consistently maintaining a higher level, as shown in Figure 5. On day 25, SOD activity in all treatments under high-temperature stress was lower than in the CK; however, SOD activity in the M1 treatment was significantly higher than in the M0 treatment, and SOD activity decreased as spray concentration increased. Regarding POD, on day 5 of treatment, POD activity under high-temperature stress was higher than that of the CK, and the M1 treatment maintained a high level; on day 15 of treatment, there were essentially no differences among treatments; on day 25 of treatment, the POD activity of the M1 treatment under high-temperature stress was higher than that of the CK. CAT activity also exhibited differences among treatments and over time. On day 5 of the treatment, CAT activity in the M0 treatment under high-temperature stress was significantly higher than that of CK; on day 15, the activity in all treatments under high-temperature stress was generally higher than that of CK, with the M1 treatment maintaining a higher level; on day 25, the activity in the M1 treatment under high-temperature stress remained at a higher level, with SD0358 showing a more significant difference compared to CK. Under high-temperature stress, MDA content was generally higher than that of the control on day 5 of treatment, with the increase being more pronounced in the M0 treatment of SD0358; on day 15 of treatment, no significant differences were observed among treatments; on day 25 of treatment, the M0 treatment generally maintained a higher level under high-temperature stress, with the MDA content of XD18 significantly elevated in the M0 treatment; XD18 exhibited an overall upward trend, while SD0358 exhibited an overall downward trend.

### 2.6. Effects of Mn_3_O_4_ Nanozymes on Wheat Yield and Yield Components Under High-Temperature Stress During the Filling Stage

Compared with the CK, the average yield of the two varieties under high-temperature stress (M0) was 21.24% lower (Table 1). The yields of all treatments involving the foliar application of Mn_3_O_4_ nanozymes were higher than those of M0, with average yield increases of 18.26%, 9.04%, and 5.34%, respectively, compared to the average yield of M0, with the M1 treatment yielding the highest average yield, which was 8.46% and 12.3% higher than that of M2 and M3, respectively. This indicates that spraying Mn_3_O_4_ nanozymes under high-temperature stress can, to some extent, mitigate the impact of high temperatures on yield; however, its effectiveness gradually diminishes as concentration increases. High-temperature stress also affects thousand-kernel weight; under high-temperature stress, the average thousand-kernel weight of M0 was 4.86% lower than that of CK, while the application of Mn_3_O_4_ nanozymes under high-temperature stress mitigated this effect, with the average 1000-grain weight increasing by 2.62%, 1.93%, and 0.79% compared to M0, respectively. Treatment effectiveness gradually diminished as concentration increased; however, Mn_3_O_4_ nanozyme treatments showed no significant differences in 1000-grain weight. The number of grains per spike and the number of spikes decreased under high-temperature stress; Mn_3_O_4_ nanozyme application showed a positive effect, but differences among treatments were not significant.

### 2.7. Correlation Analysis

**Figure 6:**
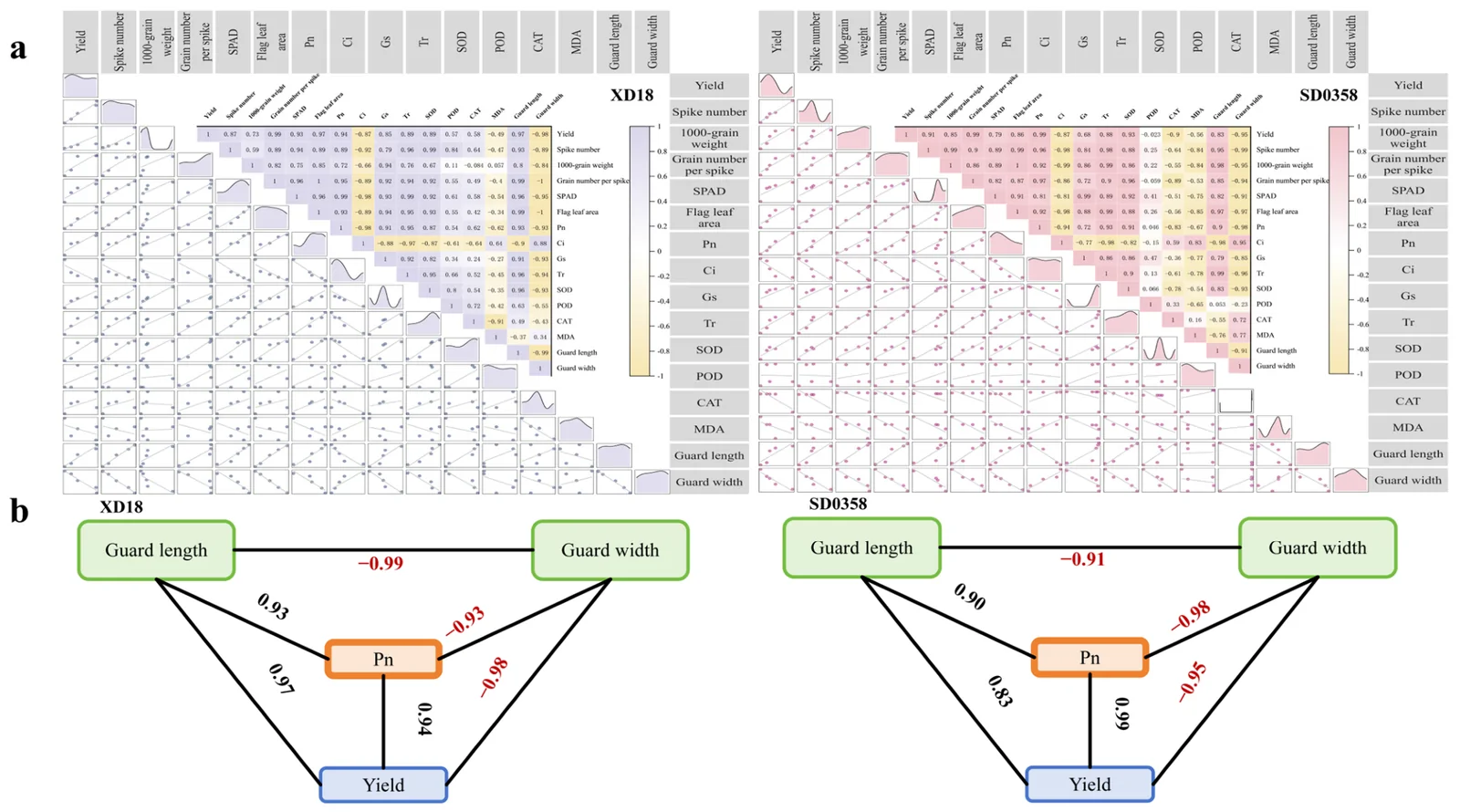
Correlation analysis of various wheat parameters (**a**) and analysis of the direct and indirect effects of guard cell morphology and photosynthetic rate on yield using PLS-SEM (**b**). The GOF value of this research model is 0.974.

As shown in the correlation analysis in Figure 6a, yield was positively correlated with thousand-kernel weight, grains per spike, number of productive spikes, and photosynthetic physiological indicators (flag leaf area, SPAD, Pn), indicating that yield formation depends not only on increases in yield-determining factors but is also closely related to photosynthetic source capacity. Further analysis revealed that SPAD and photosynthetic parameters were positively correlated with certain antioxidant enzymes, suggesting that higher antioxidant enzyme activity can more effectively scavenge reactive oxygen species, thereby delaying leaf senescence and maintaining normal photosynthetic capacity. In addition, Pn was negatively correlated with guard cell width and positively correlated with guard cell length. Furthermore, guard cell length showed a very strong positive correlation with both Pn and yield, with mean correlation coefficients of 0.92 and 0.90, respectively. In contrast, guard cell width showed a significant negative correlation with both Pn and yield, with mean correlation coefficients of −0.96 and −0.97 (Figure 6b). This indicates that, under the experimental conditions of this study, the oblong shape of guard cells may be more conducive to photosynthesis. Therefore, under high-temperature stress, spraying with Mn_3_O_4_ nanozymes can help maintain stomatal morphology and enhance antioxidant enzyme activity, ensuring normal photosynthesis and thereby stabilizing yield formation.

## 3. Discussion

### 3.1. Effects of Mn_3_O_4_ Nanozymes on Wheat Cell Structure and Stomata Under High-Temperature Stress During the Filling Stage

Chloroplasts are the primary site of photosynthesis in plants and also serve as the central site for various metabolic processes. Chloroplast damage can directly reduce photosynthetic efficiency, thereby affecting plant growth and yield [26,27,28]. Under high-temperature stress, the chloroplast membrane system is prone to damage and becomes less stable; in particular, the stacked structure of the thylakoid membranes tends to become less compact, and membrane integrity is reduced, thereby affecting photosynthetic efficiency and plant growth [29,30]. The intact, well-organized structure of chloroplasts ensures the normal and efficient conversion of light energy into chemical energy, helping wheat respond to high temperatures. In this study, representative transmission electron microscopy (TEM) observations revealed that under high-temperature stress, chloroplast structure was severely disrupted. Damaged chloroplasts were deformed and swollen, with a loose thylakoid stack structure; typically oval chloroplasts became spherical and dissolved, along with membrane structures, at the edges of the mesophyll cells. This high-temperature-induced chloroplast damage is consistent with previous research [31]. Under high-temperature stress, plants in the M1 treatment showed more stable chloroplast structures than those in other treatments; the thylakoid stack remained compact, membrane thickness was uniform, and the photosynthetic membrane system showed no significant damage, helping maintain photosynthesis under high-temperature conditions.

For plants, maintaining optimal leaf temperature and water status is key to efficient energy exchange, and stomata in the leaf epidermis primarily control this regulatory process [32]. As the key channels through which leaves exchange gases with the external environment, stomata directly affect the efficiency of photosynthesis and transpiration by regulating stomatal morphology and opening and closing behavior [33,34]. Changes in stomatal morphology under high-temperature stress primarily reflect stress-induced disruption of photosynthesis in wheat. Song et al. [35] found that high temperatures can limit stomatal expansion by regulating microtubule reorganization in guard cells, thereby reducing stomatal aperture under high-temperature stress. Furthermore, high-temperature exposure during the grain-filling stage is often accompanied by decreased leaf water potential and increased abscisic acid (ABA) levels. ABA not only induces stomatal closure but also inhibits the division and expansion of guard cells and affects stomatal size [6,36]. In this study, scanning electron microscopy (SEM) observations of stomatal morphology revealed that high-temperature stress significantly altered guard cell morphology in wheat flag leaves by reducing guard cell length and markedly increasing their width. Furthermore, under high-temperature stress, stomata remained persistently closed, whereas those treated with 100 mg·L^−1^ Mn_3_O_4_ nanozymes showed better stomatal opening. This may indicate that CO_2_ can enter the leaves unimpeded under these conditions, allowing the light and dark reactions of photosynthesis to proceed relatively normally. Furthermore, by maintaining stomatal opening, transpiration promotes water loss, which carries away a large amount of heat from the leaf surface, thereby lowering leaf temperature and reducing thermal damage to photosynthetic proteins (such as Rubisco) and membrane systems.

### 3.2. Effects of Mn_3_O_4_ Nanozymes on the Senescence of Wheat Flag Leaves Under High-Temperature Stress During the Filling Stage

The assimilates leaves produce through photosynthesis are a key source of wheat yield, with flag leaves contributing most to grain production [37,38]. As the primary organs for photosynthesis, leaves undergo accelerated senescence under high-temperature stress—a typical physiological response—thereby reducing photosynthetic capacity. A finely regulated developmental program governs leaf senescence and significantly affects plant growth, development, and yield [39]. Studies have shown that under high-temperature stress, the photosynthetic rate, chlorophyll content, and leaf area index (LAI) of wheat leaves decline, significantly reducing photosynthetic capacity [40,41]. However, applying exogenous regulators can alleviate the effects of high-temperature stress on flag leaves, slow declines in SPAD and leaf area, and improve photosynthetic capacity. Jing et al. [42] found that spraying exogenous Spm and Spd under high-temperature stress could increase net photosynthetic rate (Pn) and chlorophyll content in wheat flag leaves. Li et al. [43] found that under high-temperature stress, both green leaf area and relative chlorophyll content (SPAD) decreased significantly, whereas spraying 15 mmol·L^−1^ trehalose effectively mitigated this decline. This study found that, compared with the control group, high-temperature stress significantly reduced SPAD value, flag leaf area, and net photosynthetic rate (Pn), while increasing intercellular CO_2_ concentration. The decrease in Pn accompanied by elevated Ci indicates that under high-temperature conditions, reduced Rubisco activase (RCA) activity lowers Rubisco activation status; concurrently, impaired electron transport mediated by PSII and PSI, along with reduced Calvin cycle activity, directly damages photosynthetic organs. Coupled with restricted Ribulose-1,5-bisphosphate (RuBP) regeneration, these factors collectively reduce the CO_2_ assimilation capacity of mesophyll cells, resulting in CO_2_ accumulation in the intercellular space and ultimately increasing Ci. Additionally, reduced stomatal conductance (Gs) decreases the efficiency of CO_2_ transport from the intercellular space to the chloroplasts, further contributing to elevated Ci [44,45,46]. The application of nanozymes can effectively reverse this trend, with Mn_3_O_4_ nanoenzymes at a concentration of 100 mg·L^−1^ exhibiting superior efficacy.

As wheat enters the late grain-filling stage, the leaves gradually senesce. Under adverse conditions, an imbalance in reactive oxygen species metabolism reduces antioxidant enzyme activity and increases membrane lipid peroxide accumulation, which in turn causes grain filling to end prematurely [47,48]. Abasi et al. [49] found that under high-temperature stress, selenium-iron (SeFe) nanocomposites can effectively enhance the plant’s intrinsic antioxidant defense system, thereby alleviating oxidative damage. Consequently, the activity of enzymes in the enzymatic antioxidant system—such as superoxide dismutase (SOD), peroxidase (POD), and catalase (CAT)—is significantly enhanced. Studies have shown that the use of selenium nanoparticles (SeNPs) significantly increases CAT and SOD activities in wheat, improves photosynthetic rates, gas exchange, and transpiration rates, increases the expression levels of stress-responsive genes, enhances wheat heat tolerance, and promotes a decrease in reactive oxygen species (ROS) levels [50]. In this study, applying Mn_3_O_4_ nanozymes under high-temperature stress significantly enhanced SOD and POD enzymatic activities, which declined as treatment duration increased, consistent with patterns observed in previous studies. Mn_3_O_4_ nanozymes also significantly enhanced CAT activity, which increased over prolonged treatment durations—a finding that differs from previous research. This discrepancy may be attributed to the duration of stress exposure, the genotype of the test cultivars, or improper experimental procedures, as demonstrated in related studies. In the initial phase of thermal stress, CAT activity increases; however, by day 10, the changes in CAT activity diverge among different cultivars (Chamran and Aflak reported increases of 32% and 45%, respectively, whereas Darab2 exhibited a decrease of 22%), indicating that the ultimate CAT activity depends on both the duration of stress exposure and the cultivar genotype [51]. Furthermore, issues encountered during the operational process may also account for discrepancies in the observed activity trends. Malondialdehyde (MDA) is a product of lipid peroxidation in cell membranes; under abiotic stress conditions, it accumulates in large quantities within plants [11,52]. In this study, the MDA content in XD18 increased with prolonged stress duration, consistent with previous findings; conversely, the MDA content in SD0358 decreased. This difference may reflect variations in heat tolerance among cultivars or human-induced factors. In heat-tolerant cultivars, the antioxidant enzyme systems may effectively suppress MDA accumulation during the initial phase of stress; however, as the duration of treatment increases and the stress intensity exceeds their defense threshold, the rate of ROS generation eventually surpasses their scavenging capacity, leading to a subsequent rise in MDA levels [53]. This phenomenon reflects a delayed failure of the defense system. In contrast, prolonged treatment in heat-sensitive cultivars can cause severe membrane lipid peroxidation, potentially depleting the pool of oxidizable polyunsaturated fatty acid substrates. This depletion may reduce the rate of MDA production and significantly disrupt cellular architecture [54]. Additionally, experimental artifacts from procedural manipulations may explain discrepancies in observed activity trends—for instance, peak MDA levels in sensitive cultivars may occur before or after sampling. Furthermore, this study also found that as the treatment concentration increased, the activity of the related enzymes gradually decreased—a phenomenon that may be associated with the toxic effects at high concentrations: firstly, excessive amounts of nanomaterials themselves can act as a stress factor, potentially triggering plant defense responses and depleting the energy and metabolic resources normally allocated to defense against high-temperature stress; on the other hand, high concentrations of nanomaterials in the foliar environment may accelerate the release of Mn^2+^/Mn^3+^ ions, and the excess of free Mn^2+^ can disrupt the cofactor balance of other metalloenzymes within plant cells, potentially exacerbate oxidative damage via the Fenton reaction, thereby compromising cellular redox homeostasis [55,56,57]. In summary, the Mn_3_O_4_ nanozymes effectively alleviated the inhibitory effects of high temperatures during the grain-filling stage on wheat growth and development by slowing the decline in SPAD values, slowing the rate of decline in flag leaf area, enhancing antioxidant enzyme activity, and ultimately improving photosynthetic capacity, thereby providing a physiological basis for grain filling. These findings provide experimental evidence for further elucidating the physiological mechanisms by which Mn_3_O_4_ nanozymes enhance crop heat tolerance.

### 3.3. Effects of Mn_3_O_4_ Nanozymes on Wheat Yield Under High-Temperature Stress During the Filling Stage

Crop growth and development are regulated not only by intrinsic genetic factors but also by external biotic and abiotic factors. The grain-filling stage is a critical period for wheat grain yield formation, during which assimilates produced by the flag leaf through photosynthesis are continuously transported to the grains, directly affecting the final grain weight and yield. If wheat is subjected to high-temperature stress during this stage, yield decreases significantly [58,59]. Wheat yield components include spike number per unit area, grains per spike, and thousand-grain weight. Studies have shown that high-temperature stress negatively affects wheat grain weight, thereby decreasing yield [60]. This study found that high-temperature stress significantly affects wheat thousand-kernel weight and, consequently, yield, consistent with previous research [61]. This is because the grain-filling stage is highly sensitive to temperature changes; high-temperature stress reduces the number of endosperm cells in the grain, inhibiting endosperm cell proliferation and decreasing storage capacity [62,63,64]. Abasi et al. [49] found that treatment with selenium/iron nanocomposites during the highly heat-sensitive stage of flowering significantly enhanced wheat’s antioxidant defense mechanisms, thereby improving wheat yield and quality under high-temperature stress. Iqbal et al. [65] also found that synthesized AgNPs can effectively enhance wheat tolerance to high-temperature stress by improving physiological and biochemical conditions and activating the antioxidant defense system. In this study, applying Mn_3_O_4_ nanozymes under high-temperature stress alleviated the inhibitory effect of high-temperature stress on yield, with 100 mg·L^−1^ Mn_3_O_4_ nanozymes showing the greatest alleviating effect.

However, this study still has certain limitations. First, the high-temperature stress treatment in this research was conducted under greenhouse conditions, which differs from natural extreme high temperatures in the field. Second, this study used only two wheat cultivars, limiting the generalizability of the findings to different genotypes. Future studies should incorporate a broader range of cultivars to validate the universality of the observed effects. Third, this study did not investigate the foliar absorption, transport, or accumulation of Mn_3_O_4_ nanozymes in plant tissues. Although the physiological and biochemical effects of these nanozymes have been clearly demonstrated, the specific pathways and efficiency of their foliar penetration, as well as the distribution and fate of these nanomaterials within plants, remain unclear. Future work should use tracer techniques to quantitatively assess nanozyme absorption and transport, which would be crucial for optimizing application protocols and evaluating environmental safety. One limitation is the lack of measurements of solution pH, ROS accumulation, membrane damage, Mn uptake, and nanozyme localization. Consequently, the mechanism of action of Mn_3_O_4_ nanozymes remains inferential rather than directly demonstrated. In this study, high concentrations of Mn_3_O_4_ nanozymes may have toxic effects on wheat, thereby suppressing its physiological traits; however, the study did not quantify Mn accumulation or other toxicity-related indicators. Future research should assess Mn uptake and determine toxicity biomarkers to clarify whether the diminished regulatory effects at high concentrations stem from plant toxicity.

## 4. Materials and Methods

### 4.1. Site Description

The experiment was conducted from September 2024 to June 2025 at the Manas Experimental Station of the Xinjiang Academy of Agricultural Sciences (N 44°18′, E 86°13′). The region has a temperate continental climate. During the winter wheat growing season in this experiment, the average temperature was 5.74 °C, with total precipitation of 3.14 mm. During the high-temperature treatment period in 2025, the average temperature was 25.65 °C, with total precipitation of 0.44 mm (Figure S1). The experimental site is flat and has sandy loam soil. Before sowing, the 0–20 cm soil layer contained 22.59 g·kg^−1^ soil organic matter, 94.06 mg·kg^−1^ alkaline nitrogen, 842.39 mg·kg^−1^ available phosphorus, and 70.45 mg·kg^−1^ available potassium.

### 4.2. Experimental Design and Setup

In this experiment, a greenhouse with artificial polyethylene plastic film was used; a high-temperature environment was established on the fifth day after flowering and maintained until day 28 post-flowering, for a total high-temperature stress duration of 23 days. The greenhouses were 1.5 m high, with lengths and widths matched to the experimental plot area. Ventilation holes were installed at the base of the greenhouses to keep the average temperature within the wheat canopy 2–3 °C higher than it would be under natural conditions.

The experiment employed a single-factor randomized block design. Five treatments were established: Under high-temperature conditions, Mn_3_O_4_ nanozymes at varying concentrations were sprayed in combination with Silwet L-77 to counteract the hydrophobicity of the leaf cuticle—using distilled water (M0),100 mg·L^−1^ (M1),150 mg·L^−1^ (M2), and 200 mg·L^−1^ (M3); as well as distilled water in a control group (CK) under natural outdoor conditions in the greenhouse, and the spray rate was 225 L·ha^−1^. Each treatment was replicated three times, with each plot measuring 4 m^2^ (2 m × 2 m). Two spray applications were conducted on windless, rain-free afternoons: on the 0th and 10th days after greenhouse construction. The treatment solutions and distilled water were evenly sprayed onto the leaves. The test varieties were Xindong 18 (XD 18) and Shidong 0358 (SD 0358). The seeding rate was 6 million seeds·ha^−1^. Before sowing, 75 kg·ha^−1^ of urea (46% pure N) and 357 kg·ha^−1^ of diammonium phosphate (46% pure P_2_O_5_, 18% pure N) were applied during land preparation. During the growing season, 360 kg·ha^−1^ of urea was applied as a foliar spray with irrigation water. Drip irrigation was used, with a total irrigation volume of 4200 m^3^·ha^−1^. All other cultivation practices were consistent across all treatments. Sowing began on 24 September 2024, and harvest occurred on 23 June 2025. Mr. Liu Jiahao of Tarim University prepared the Mn_3_O_4_ nanozymes, and their characterization data (particle size, purity, and morphology) match those reported in his previous studies [66].

### 4.3. Plant Sampling and Measurements

#### 4.3.1. Canopy Temperature and Humidity

We recorded data using a MicroLite 5032P-RH USB-style temperature and humidity data logger (Fourtec–Fourier Technologies Ltd., Rosh Haayin, Israel), which we placed in the wheat canopy after setting up the greenhouse. Recordings were taken every hour until the high-temperature treatment ended.

#### 4.3.2. Microstructure of Leaves

On day 15 after high-temperature stress treatment, wheat plants with comparable growth status were selected from each treatment group, and three flag leaves were harvested from each plant; subsequently, representative images were captured for each treatment group, and qualitative descriptions and analyses were performed on these images. After rinsing the surface dust off the flag leaves with running water, the leaves were gently blotted dry with absorbent paper. Tissue blocks measuring approximately 1 cm × 1 cm were cut from the middle of the leaves, immersed in an electron microscope fixative, and stored away from light for subsequent scanning electron microscopy (SEM, SU8100, Hitachi High-Tech Corporation, Tokyo, Japan) observation of stomatal morphology and transmission electron microscopy (TEM, HT7800, Hitachi High-Tech Corporation, Tokyo, Japan) observation of cellular structure.

#### 4.3.3. Flag Leaf Area

On days 0, 5, 10, 15, and 20 after treatment, ten wheat plants with uniform growth were selected from each treatment, and their flag leaf area was measured. Leaf area was determined using the length-to-width ratio method.

#### 4.3.4. SPAD Value

At 0, 5, 10, 15, 20, and 25 days after treatment, the relative chlorophyll content in the flag leaves of wheat under different treatments was measured using the SPAD 502 Plus chlorophyll meter (Konica Minolta, Osaka, Japan). For each plot, ten leaves were randomly selected for SPAD measurements.

#### 4.3.5. Photosynthetic Parameters

At 0, 7, 14, and 21 days, the net photosynthetic rate (Pn), intercellular CO_2_ concentration (Ci), transpiration rate (Tr), and stomatal conductance (Gs) of winter wheat flag leaves were measured using the Li−6800 portable photosynthesis system (LI-COR Biosciences, Lincoln, USA) between 10:30 and 12:00 on sunny days. Measurements were performed in a leaf chamber with a CO_2_ concentration of 400 ppm, a mean temperature of 25 °C, a flow rate of 500 µmol s^−1^, and a light level of 1600 µmol m^−2^ s^−1^. For each treatment, we randomly selected three flag leaves for measurement.

#### 4.3.6. Activities of Antioxidative Enzymes

On days 5, 15, and 25 after treatment, we collected fresh flag leaves from wheat plants with uniform growth in the field. The leaves were wrapped in aluminum foil, labeled, and rapidly frozen in liquid nitrogen, then stored at −80 °C. The Boxbio kit (Beijing Boxbio Science & Technology Co., Ltd., Beijing, China) was used to determine malondialdehyde (MDA) content and the activities of superoxide dismutase (SOD), catalase (CAT), and peroxidase (POD).

#### 4.3.7. Yield and Its Components

During the wheat maturity stage, representative 1 m × 2-row plots were selected from each experimental block for indoor evaluation, and the number of effective spikes (3 replicates), grains per ear (30 replicates), and thousand-grain weight (15 replicates) were measured. The harvested area of each plot (actual harvest area 3.2 m^2^) was used to determine yield with 3 replicates.

### 4.4. Data Statistics and Analysis

Data were processed in Microsoft Excel 2016, and statistical significance was determined using Tukey’s honest significant difference (HSD) test in one-way analysis of variance (ANOVA). For yield, a two-way ANOVA (variety × treatment group) was employed. Data were analyzed in SPSS Statistics 27 (IBM, Chicago, IL, USA). Guard cell length and width were measured using ImageJ-win64. Figures were created using Origin 2025, while correlation plots were constructed with the ‘ggplot2’ and ‘GGally’ packages in R 4.6.0 and Partial Least Squares Structural Equation Modeling (PLS-SEM) using ‘plspm’ and ‘qgraph’.

## 5. Conclusions

High-temperature stress during the grain-filling stage not only damages chloroplast membranes and disrupts thylakoid membrane stacking but also significantly inhibits stomatal opening, reducing flag leaf area, SPAD, and Pn. This weakens photosynthetic capacity and ultimately reduces yields. Exogenous application of Mn_3_O_4_ nanozymes effectively alleviated stress damage. Its mechanism of action involved maintaining chloroplast ultrastructure and stomatal aperture, delaying declines in flag leaf area and SPAD values, and slowing the reduction in photosynthetic capacity. At the same time, it significantly enhanced antioxidant enzyme activity; this synergistic effect between photosynthesis and antioxidant physiology ultimately improved yield. Among the treatments, the 100 mg·L^−1^ Mn_3_O_4_ nanozymes treatment yielded the best results, with an average yield increase of 18.26% compared to the distilled water control (M0) under high-temperature stress; simultaneously, compared to M0, this mitigating effect showed a decreasing trend as the spray concentration increased, resulting in yield increases of 9.04% and 5.34%, respectively, compared to M0. This indicates that an appropriate concentration of Mn_3_O_4_ nanozymes is key to exerting a protective effect, which can serve as a practical reference for stress-tolerant, stable-yield cultivation. However, the high-temperature stress conditions simulated in greenhouses differ from those in natural extreme high-temperature environments; therefore, this study’s conclusions apply only to simulated greenhouse conditions and should not be directly extrapolated to natural field environments under extreme high-temperature conditions.

## Figures and Tables

**Table 1:** Effects of spraying Mn_3_O_4_ nanozymes on wheat yield and its components under high-temperature stress during the grain-filling stage.

Cultivar	Treatment	Spike Number (×10^4^·ha^−1^)	Grain Number per Spike	Grain Weight (g)	Yield (kg·ha^−1^)
XD18	CK	645.71 ± 3.21 a	39.53 ± 10.49 a	42.32 ± 0.30 a	8021.82 ± 10.06 a
	M0	641.82 ± 0.68 a	36.33 ± 6.05 a	39.90 ± 1.16 b	6584.77 ± 15.49 d
	M1	645.70 ± 13.37 a	38.90 ± 6.54 a	40.41 ± 0.69 b	7992.29 ± 18.89 a
	M2	645.56 ± 4.72 a	37.70 ± 4.85 a	40.33 ± 0.08 b	7166.46 ± 29.73 b
	M3	642.93 ± 6.31 a	36.90 ± 6.72 a	40.06 ± 0.67 b	6874.95 ± 25.69 c
SD0358	CK	533.99 ± 3.15 a	52.90 ± 8.82 a	43.39 ± 0.01 a	9543.99 ± 45.30 a
	M0	530.19 ± 3.61 a	51.07 ± 1.98 a	41.66 ± 1.37 c	7199.91 ± 15.96 e
	M1	533.51 ± 0.96 a	51.90 ± 7.48 a	43.30 ± 0.32 a	8289.10 ± 28.62 b
	M2	532.13 ± 1.76 a	51.80 ± 6.92 a	42.82 ± 0.12 ab	7864.97 ± 30.53 c
	M3	531.36 ± 1.36 a	51.50 ± 3.31 a	42.15 ± 0.67 bc	7650.53 ± 35.68 d
Cultivar		**	**	**	**
Treatment		ns	ns	**	**
Cultivar × Treatment		ns	ns	**	**

Note: The data in the table are expressed as mean ± standard deviation. Different lowercase letters within the same column indicate statistically significant differences among treatments. The significance markers for cultivar, treatment, and interaction effects are as follows: ns, not significant (*p* > 0.05); **, *p* < 0.01.

## Data Availability

All data supporting the findings of this study were included within the manuscript.

## Supplementary Materials

The following supporting information can be downloaded at: https://www.mdpi.com/article/10.3390/plants15203096/s1, Figure S1: Field trial setup (a,b); Precipitation and average temperature in trial plots during the 2024–2025 growing season (c); Hourly temperature variations inside and outside the greenhouse during high-temperature stress (d); Daily average temperature variations inside and outside the greenhouse, as well as indoor humidity, during high-temperature stress (e); Figure S2: Correlation analysis of all indicators for XD18 (a); correlation analysis of all indicators for SD0358 (b).

## Abbreviations

The following abbreviations are used in this manuscript:

ABA	abscisic acid
CAT	catalase
Ci	intercellular CO_2_ concentration
Gs	stomatal conductance
MDA	malondialdehyde
Pn	net photosynthetic rate
POD	peroxidase
ROS	reactive oxygen species
SEM	scanning electron microscopy
SOD	superoxide dismutase
TEM	Transmission electron microscopy
Tr	transpiration rate
