# Supplementary material for: Effects of Spraying Mn3O4 Nanozymes at Different Concentrations on the Physiological Characteristics and Yield of Wheat Flag Leaves Under High-Temperature Stress During the Filling Stage
Source: Plants (Basel). 2026 Oct 10;15(20):3096. doi: 10.3390/plants15203096 (PMC13709275; doi:10.3390/plants15203096)
Supplement: Supplementary file 1 [file plants-15-03096-s001.zip › plants-4586127-supplementary.pdf]

SUPPLEMENTARY MATERIAL

Effects of Spraying Mn_3O_4 Nanozymes at Different Concentrations on the Physiological Characteristics and Yield of Wheat Flag Leaves Under High-Temperature Stress During the Filling Stage

Qian Yang [1], Chuanxin Chen [2], Junjie Lei [2], Shihui Nie [2], Juan Li [2], Yuting Hou [2], Wenxiu Xu [1,] and Yongqiang Zhang [2,*]*

1 College of Agriculture, Xinjiang Agricultural University, Urumqi 830052, China; 17361238862@163.com

2 Institute of Crop Research, Xinjiang Academy of Agricultural Sciences, Key Laboratory of Desert–Oasis Crop Physiology, Ecology and Cultivation, Urumqi 830091, China; lixp618@163.com (C.C.); leijunjie@sohu.com (J.L.); nieshihui@hotmail.com (S.N.); l_juan@yeah.net (J.L.); yuthou@163.com (Y.H.)

* Correspondence: xjxuwenxiu@163.com (W.X.); zyq988@yeah.net (Y.Z.)

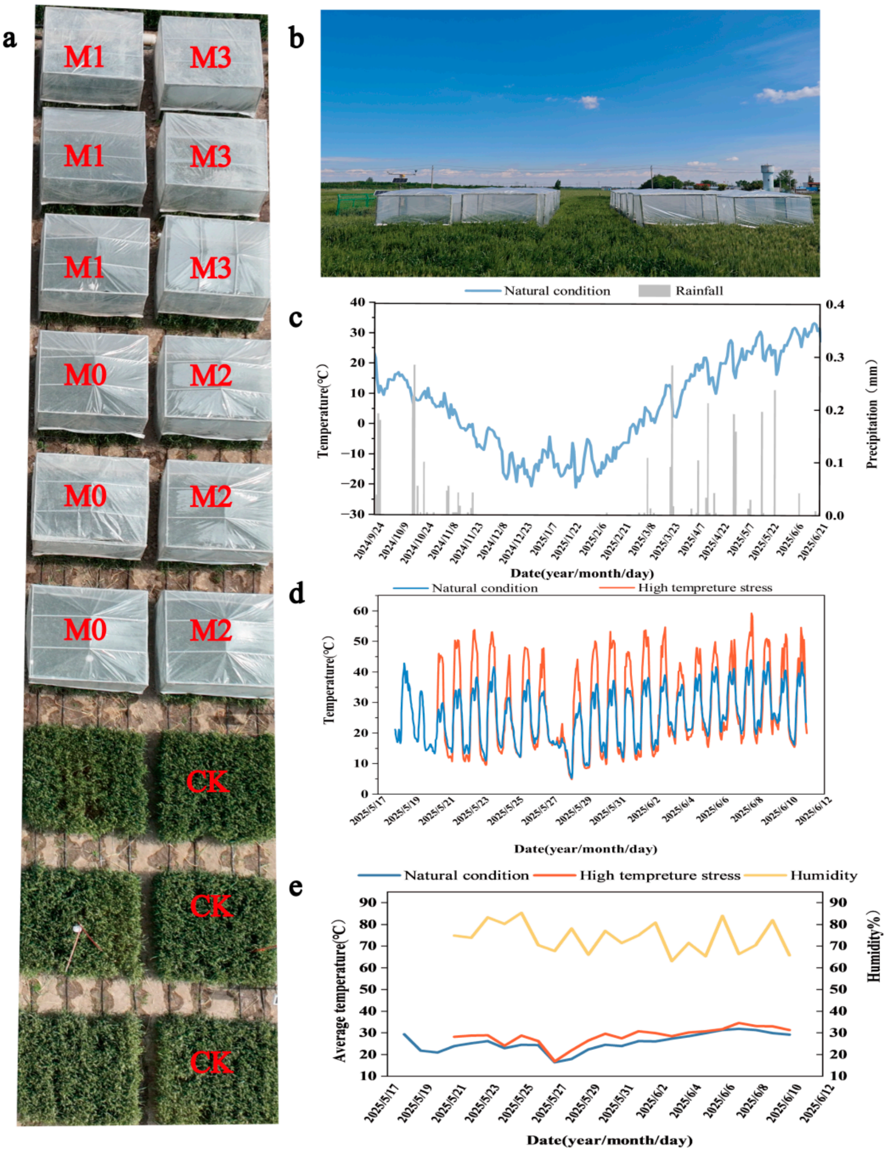

Figure S1. Field trial setup (a, b); Precipitation and average temperature in trial plots during the 2024 - 2025 growing season (c); Hourly temperature variations inside and outside the greenhouse during high-temperature stress (d); Daily average temperature variations inside and outside the greenhouse, as well as indoor humidity, during high-temperature stress (e).

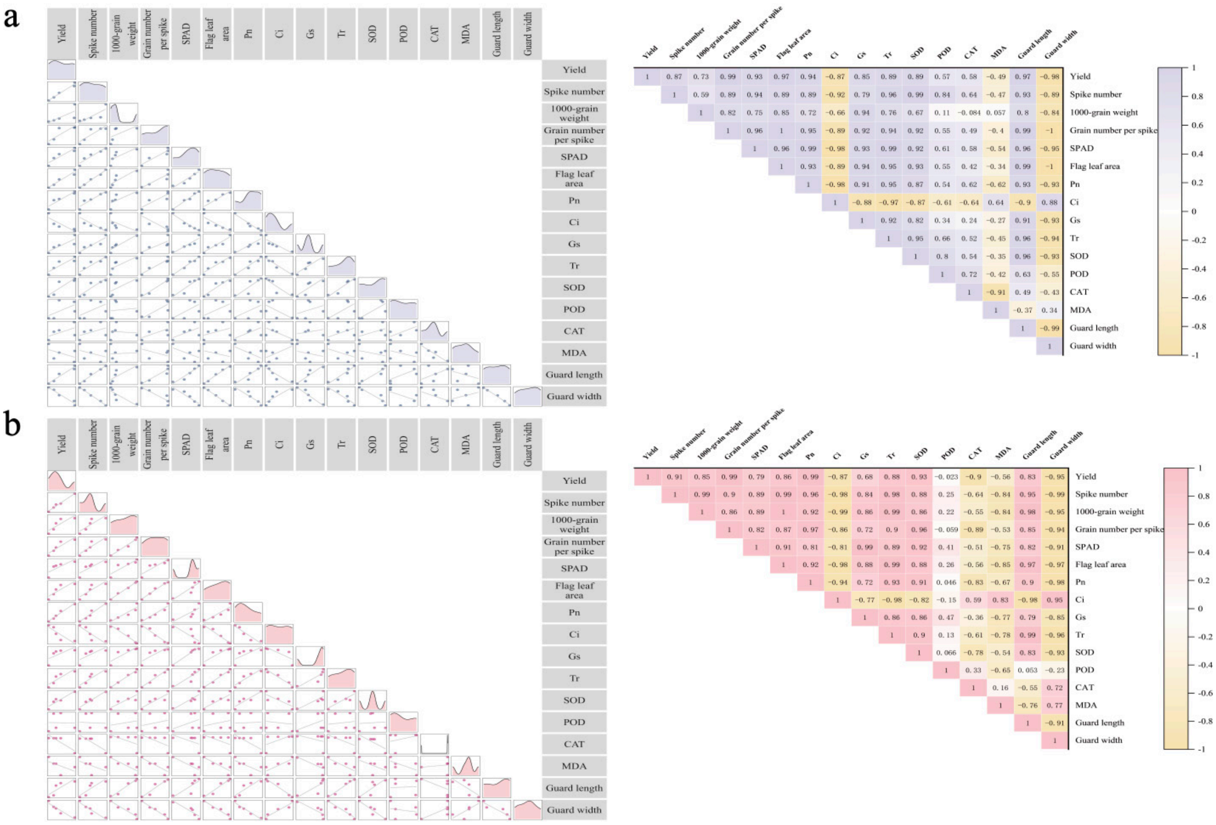

Figure S2. Correlation analysis of all indicators for XD18(a); correlation analysis of all indicators for SD0358 (b).
